# Functional activities of beta-glucans in the prevention or treatment of cervical cancer

**DOI:** 10.1186/s13048-020-00626-7

**Published:** 2020-03-05

**Authors:** Shahla Chaichian, Bahram Moazzami, Fatemeh Sadoughi, Hamed Haddad Kashani, Marsa Zaroudi, Zatollah Asemi

**Affiliations:** 1 Pars Advanced and Minimally Invasive Medical Manners Research Center, Pars Hospital, Iran University of Medical Sciences, Tehran, Iran; 2 Research Center for Biochemistry and Nutrition in Metabolic Diseases, Kashan University of Medical Sciences, Kashan, I.R Iran; 3 Anatomical Sciences Research Center, Kashan University of Medical Sciences, Kashan, Iran; 4 Student Research Committee, Faculty of Public Health Branch, Iran University of Medical Sciences, Tehran, Iran

**Keywords:** Beta-glucans, Cervical cancer, Anti-cancer, Treatment, Prevention, Sizofiran

## Abstract

Cervical cancer is the fourth-ranked cancer in the world and is associated with a large number of deaths annually. Chemotherapy and radiotherapy are known as the common therapeutic approaches in the treatment of cervical cancer, but because of their side effects and toxicity, researchers are trying to discovery alternative therapies. Beta-glucans, a group of glucose polymers that are derived from the cell wall of fungi, bacteria, and etc. it has been showed that beta-glucans have some anti-cancer properties which due to their impacts on adaptive and innate immunity. Along to these impacts, these molecules could be used as drug carriers. In this regard, the application of beta-glucans is a promising therapeutic option for the cancer prevention and treatment especially for cervical cancer. Herein, we have summarized the therapeutic potential of beta-glucans alone or as adjuvant therapy in the treatment of cervical cancer. Moreover, we highlighted beta-glucans as drug carriers for preventive and therapeutic purposes.

## Introduction

Cervical cancer is known as the fourth cancer among women and the eighth common cancer worldwide [1, 2]. Cervical cancer is a result of being infected by some types of human papillomavirus or HPV, which are effective on the mucosa [3, 4]. Findings indicated that HPV is a necessary cause of cervical cancer; this means that the other risk factors can increase the risk of cervical cancer, but they are not able to develop this kind of cancer in the absence of HPV [5]. In this regard, potential cofactors involved in the cervical cancer pathogenesis are divided into three classes: 1) Environmental risk factors such as using hormonal birth control [6, 7], smoking [8], parity (the number of sex partners) [9], and have a history of being infected with other sexually transmitted agents [10–12]. 2) Viral risk factors including having a history of being infected with other HPV types, viral load, and integration of several viruses [13]. 3) Host risk factors like endogenous hormones, genetic-related factors, and generally any factor that can affect on the response of the immune system [13].

It has been indicated that a variety of therapies could be used in the treatment of different cancers such as cervical cancer. In this word, many therapeutic approaches i.e., surgery, chemo, and radiotherapy, utilization of immune checkpoint inhibitors, therapeutic vaccines, and antibody-drug conjugates, gene- and cell-based therapy, and nanotechnology-based therapies could be employed as primary lines of therapy in the cancer therapy [14–20]. Given that some of the above approaches such as surgery, chemo and radiotherapy, vaccine- and antibody-based therapies have therapeutic candidates in the cervical cancer therapy [21].

Glucans are a kind of polymers that are made up of glucose monomers alone and are known as a heterogeneous class of polysaccharides. These glucose polymers are different from each other in some properties such as their chain length, having branches, being alpha or beta isomers and their ability to dissolve [22]. Beta-glucans can be found in different plants such as oat, barley, and seaweed. In addition, they are also involved in fungal and pathogenic bacterial cell walls [23]. Cellulose, curdlan, laminarin, chrysolaminarian, lentinan, lichenin, pleuran, zymosan, and schizophyllan are some examples of beta-glucans (Table 1) [60]. These glucose polymers could be linked together by β (1 → 3) linkages and provide the linear β-glycosidic chain core [61]. The glycosidic core could provide different kinds of branches. Two important groups of them are 1 → 4 or 1 → 6 glycosidic chains [62]. Diverse structures of beta-glucans can proceed from different resources. For instance, beta-glucans derived from oat and barley are linear with large regions of beta (1, 4) linkages separating shorter stretches of beta (1, 3) structures while beta-glucans of mushrooms have short branches with beta (1, 6)-link coming off of the beta (1,3) backbone. Yeast beta-glucans have beta (1, 6) branches that are further intricate with additional beta (1, 3) regions. These diversities in the beta-glucan structures are the reason why they have different functions. The biological functions of beta-glucans depend on their interaction with their receptors. These interactions are also dependent on two factors including conformation of these molecules and their water solubility. It is acknowledged that the water-soluble glucans are more efficient [63].

**Table 1:** Different beta-glucans, their characteristics, sources, and potential uses

Name of beta-glucan	Type	Source	Characteristic (s)	Potential application (s)	References
Cellulose	Beta-1,3-glucan	Plant and bacterium (Acetobacter species)	Insoluble in water, straight-chain, high mechanical strength,	Wound-dressing	[24–28]
Curdlan	Beta-1,4-glucan	Bacterium (Agrobacterium species)	Water-insoluble, linear, high molecular weight, affecting innate and adaptive immunities, easy modification	Immunological functions and drug delivery	[29–33]
Laminarin	Beta-1,3 and beta-1,6-glucan	Brown algae	Low molecular weight, linear, low viscosity, antioxidant, and antimicrobial activities	Development of a new injectable system	[34–37]
Chrysolaminarian	Beta-1,3-glucan	Microalgae	Linear, antioxidant and immunomodulatory capacity suitable for immunostimulation	Development of a new injectable	[38, 39]
Lentinan	Beta-1,3 and beta-1,6-glucan	Fungus (Shiitake)	Affecting T helpers and macrophage, non-toxic and well-tolerated immunomodulator, initiating an inflammatory response	Anti-tumor effects with low side effects	[40–43]
Lichenin	Beta-1,3 and beta-1,4-glucan	Lichens (*Cetraria islandica*)	Linear, non-toxic, stimulating effects on T cells	Being used as an Anti-tumor agent	[44–46]
Pleuran	Beta-1,3 and beta-1,6-glucan	Fungus (Pleurotus ostreatus)	Immunomodulatory and anti-inflammatory activity	Treatment of dermatitis, being used as an anti-cancer and anti-allergic agent	[47–51]
Zymosan	Beta-1,3-glucan	Yeast	Anti-inflammatory effects, affecting dendritic and T cells, affecting cytokine expression	Being used as treatment for several diseases such as cancer, peritonitis and etc.	[52–55]
Schizophyllan	Beta-1,3 and beta-1,6-glucan	Fungus (Athelia rolfsii)	Stimulate the immune system, carry metals in water	Aid in delivering drugs and genes, and use in some nanofibers	[56–59]

Some in vitro experiments have demonstrated that large beta-glucans or some of their particular ones (like zymosan) can cause activation of leukocytes and stimulate some of their activities such as phagocytosis, cytotoxicity, and antimicrobial actions. Intermediate or low molecular weight beta-glucans such as glucan phosphate can have biological activities in vivo, but still, their influence on cancer cells is unclear. Very short b-glucans like laminarin are generally believed to be inactive [64–66]. In general, some functions are attributed to these polymers: they can have an activate macrophages and thereby increase the secretion of cytokines such as interleukin 1 (IL-1), IL-6, IL-8, IL-12, and tumor necrosis factor-α (TNF-α) and inflammatory mediators [67]. Therefore, beta-glucans can influence on immune defense system. Moreover, investigations on the plasma amounts of beta-glucans showed that they can be involved in the mammal’s defense mechanism against the fungal infections [68, 69]. Apart from activating macrophages, T cells, and natural killer cells, and complement can be activated by beta-glucans by the means of an alternative activation pathway, as well [70]. Beside the impacts of beta-glucans on immune system, beta-glucans are able to decrease the levels of insulin, blood sugar, and cholesterol by remaining inside the colon and intestine (oat and barley beta-glucans) [60]. Recently, new studies are have assessed the effects of these polymers on tumor cells and they suggested that beta-glucans can be proper candidates in the cancer therapy due to two reasons including their anti-cancer activities and immune system modulatory roles [23]. This review is an attempt to find an answer to this question: “do beta-glucans have any impact on cervical cancer?”

### How do beta-glucans function?

The focus of recent researches has been on the interaction between beta-glucans and their receptors, which are located on the cell surface, and how they allow these polymers to affect on the cells and make some alterations. Generally, glucan receptors can be expressed on macrophages, natural killer cells and neutrophils [23], however monocytes are the first cells in which beta-glucan receptors were found [71]. There are four different receptors for beta-glucans including complement receptor 3 or CR3 [72], lactosylceramide [73], selected scavenger receptors [74], and dectin-1 (bGR) [75]. The CR3 is a glycoprotein dimer and consists of two subunits: a CD18 beta chain, which is noncovalently linked to one type of three alpha chains (which can be CD11a, b or c). This glycoprotein is a member of the beta integrin family and acts as a transmembrane glycoprotein. CR3 is mostly found on the plasma membrane of the phagocytic cells like neutrophils and NK cells, monocytes and less on macrophages. Functions of CR3 can be different in its resting or activated state but mediating phagocytosis, cytotoxic reactions, and cellular adhesion are counted as its main functions [76].

Lactosylceramide is a kind of glycosphingolipid which is expressed on the membranes of many cells of the body. This receptor has many functions including induction of the inflammatory protein in the macrophages (called MIP-2), activation of *nuclear factor-κB* (NF-kB), improvement of oxidative stress in the neutrophils and some anti-microbial activities. Although the real mechanisms of these receptors are still unclear [73]. Selected scavenger receptors are a part of predetermined pattern recognition receptors (PRRs) that in spite of the adaptive immunity do not identify the host products. They are involved in the innate immune system and are used to recognize the microorganisms by their unique carbohydrates, lipids, and proteins. Dectin-1 or beta-glucan receptor (bGR) is a type II transmembrane protein and it is able to attach to two kinds of glucans: β-1, 3 and β-1, 6. This receptor is mostly expressed on cells in the innate immune system and the main role of it is recognizing the yeast and fungi pathogens. The tail of this receptor consists of an immunoreceptor tyrosine-based activation motif (ITAM). Some functions are found for this receptor-like including stimulating the release of arachidonic acid, which is an important factor for producing some substances during the process of acute inflammation. Moreover, it is proven that it participates with toll-like receptor 2 and thereby induces the pro-inflammatory response of macrophages in mycobacterial infections [77–80].

### Beta-glucan and cancer

Beta-glucans have complicated structures and because of that, they have several pivotal roles in human body such as increasing resistance to infectious challenges [23], anti-carcinogenic activities [81, 82], anti-tumor effects [61], and activating leukocytes, T helpers, and NK cells [83], anticoagulant effects [84], and antibiotic impacts [85]. Generally, three kinds of study that investigated the role of beta-glucans as a modulator for the immune system:

1. In vitro studies: several studies have proven that beta-glucans can act as an enhancer for macrophages and neutrophils [86–88]. This function is possible because they can make an elevation to the production of chemokine and pro-inflammatory cytokines and can augment the oxidative burst [86, 89–91]. Olson et al. [89] revealed that *S. cerevisiae* can affect on the alveolar macrophages and cause an elevation in the amounts of TNF-α production in rats. Another study demonstrated that zymosan is able to induce the secretion of TNF-α [92]. Scientists have observed that adding the combination of soluble yeast beta-glucan and lipopolysaccharides (LPS), which are a part of pathogen-associated molecular patterns, to the whole human blood could increase the concentration of TNF-α, IL-8, IL-10, and tissue factor (TF) [93]. Additionally, these impacts are also proven for fungi, yeast, and oat beta-glucans. Estrada et al. [94] observed an augmentation in the production of IL-1a in murine macrophages because of oat beta-glucans and also detected an improvement in the secretion of IL-2, IFN-gamma, and IL-4. Augmenting the activity of leukocytes is a function of PGG or poly-[1, 6]-D-glucopyranosil-[1, 3]-D-glucoyranose which is extracted from yeast. This beta-glucan makes neutrophils migrate more toward C5a and it induces the stimulating responses of the immune system with no effects on the production of cytokines involved in inflammation [95].
  Dendritic cells are a kind of antigen-presenting cells, which Lin et al. [96] discovered the impact of fungal beta-glucan P-SG on them. They revealed that it results in stimulation in T helper − 1 type of cytokine response. Furthermore, beta-glucans not only can affect on leukocytes but they also can induce epithelial cells response. For example, pneumocystis carinii beta-glucan is able to stimulate the alveolar epithelial cells in rats to secret macrophage inflammatory protein-2 [97]. Taken together, beta-glucans modulate the response of the immune system by cytokine secretion regulating. Another in vitro study found that maitake mushroom beta-glucan can induce apoptosis in prostate cancer cells by affecting on oxidative stress. Tian et al. [98] also revealed that beta-glucans are able to abolish the myeloid-derived suppressor cells. Myeloid-derived suppressor cells or MDSCs suppress the immune system and induce tumor progression. Thus, beta-glucans via abrogating them help the anti-tumor responses of the immune system. A report also confirmed the down-regulation effect of beta-glucans on another unit that has the immunosuppressor effects on regulatory T cells (Tregs) [99].
2. Animal studies: many researchers have worked on isolated leukocytes which are extracted from an animal treated with beta-glucans. These studies, in agreement with in vitro studies, has also observed an elevation in production amounts of pro-inflammatory cytokine [100], in oxidative burst [101], and chemotaxis [102] and overall an induction in the specific immunity of type 1 T helpers and also an elevated survival rate against the infection of pathogens which can be concluded from this kind of researches [83].
3. Human studies: in spite of the numerous trials carried out in vitro and on animals, the number of human studies is limited. The only thing that these studies have shown is the decreased risk of being infected and the need for antibiotics in pre-treatment of high-risk surgical patients [103, 104]. Another trial showed that zymosan stimulates the regulatory antigen-presenting cells and as well, increases the tolerance of the antigen-specific T cells. Moreover, beta-glucans extracted from *Candida albicans* cause an induction in the differentiation of monocytes into dendritic cells [105].

### Role of beta-glucans in monoclonal antibodies (MABs)-associated immunotherapy

In recent years, a novel therapeutic method has been discovered that is an insight for replacing chemo and radiotherapy with large amounts of toxicity and side effects in cancer treatment. The preference of this approach is because of its specific action that only affects the tumor cells and therefore it minimizes the surrounding tissue injury [23]. This new method is based on the activation of complement by using monoclonal antibodies. A group of antibodies which all of them are derived from the same B-lymphocyte clone and are linked to each other is the definition of this kind of antibodies or mABs [106]. Direct destruction of tumor cells is the result of their role as an activator for the complement system. MABs direct the cell-killing impressions to a tumor cell by using three mechanisms including complement-dependent (CDC), antibody-dependent (ADCC), and CR3-dependent cytotoxicity (CR3-DCC). The CR3 is one of the beta-glucans receptors and hence, in order to be activated, it needs to be linked into a fungal or bacterial beta-glucan. Additionally, there is another ligand that binds to another site of CR3 and is essential for activating these receptors including iCR3b which is an antigen for tumor cells and coats them. Nevertheless, in spite of the microorganisms, tumor cells do not possess beta-glucans on their surface. Consequently, the exogenous beta-glucans are needed to induce this mechanism and so this method can be effective on tumor cells adjuvant with beta-glucans [107]. Some studies by administering beta-glucans orally [108] and intravenous [109] have found some significant results in this field.

### The impact of beta-glucans on myeloid-derived suppressor cells

Myeloid-derived cells are a heterogeneous population. The mononuclear type of these cells includes macrophages, dendritic cells, and monocytes which are terminally differentiated. These cells in the case of inflammation differentiate into macrophages and dendritic cells. MDSC suppresses different kinds of immunity cells especially T cells and thereby their main function is suppressing the immune system in antigen-specific or non-specific manners. MDSC includes two classes of cells: granulocytic or polymorphonuclear (PMN-MDSC) and monocytic (M-MDSC) [110]. T reg cells, myeloid suppressor cells, and inhibitory cytokines are three considerable components of the immune system which cause the destruction of anti-tumor immunity [111]. Current investigations have informed another anti-cancer property for beta-glucans including abrogating MDSC. Albeituni et al. [112] indicated that particulate beta-glucans can corrupt both PMN-MDSC and M-MDSC by stimulating the apoptosis of the former and regulating the differentiation of the latter one into APC in cancer. As well, there are some other studies confirming the part of beta-glucans are able to inhibit the differentiation of MDSC [113, 114]. Rui et al. [115] considered a beta-glucan extracted from bacteria, curdlan, and its actions against the tumor progression and observed that curdlan is able to boost the MDSCs differentiation and therefore it decreases the number of immature MDSCs. This action of curdlan results in decreasing the suppressive impact of MDSCs against anti-tumor immunity. Furthermore, another group of researchers, Ning et al. [116], worked on a beta-glucan derived from the yeast *Saccharomyces cerevisiae* named WGP beta-glucan. They found that WGP not only can activate immature dendritic cells, T-helper 1, and differentiation of the cytotoxic T lymphocytes through dectin-1 signaling but it can also abolish the immune suppression caused by tumor-educated dendritic cells and improve priming of T cells. Taken together, WGP is another important beta-glucan in promoting anti-tumor immunity.

### Developmental mechanisms of cervical cancer

As mentioned, the most crucial risk factor for cervical cancer is being infected by human papillomavirus or HPV (especially HPV 16) [117]. Till now, more than 100 types of HPV are discovered but only 40 types of them can infect the human genital area. HPV fragments include 8000 based-pair of long circular DNAs which are covered with a mantle of two proteins: L1 and L2. HPV also needs six other proteins (named E1, E2, and E4-E7) for replicating the viral DNA and the group of viral particles that are newly produced by the infected cell. Generally, HPV infects the differentiating epithelial cells of the skin or mucosae and consequently, it’s completely adjusted to its host and utilizes the host’s cellular mechanisms to achieve its own targets [13]. E6 and E7 are two kinds of viral proteins that have essential roles in the virus replication process [118] and so they can determine the risk of HPV types [119]. These two proteins exert their functions via interaction with some cellular proteins such as p53 and pRB (retinoblastoma protein, a tumor suppressor). P53 and pRB are central molecules for regulating the cell cycle and hence their mutation can have important roles in the initiation and progression of various diseases such as cancer [118]. The binding of E6 to p53 provides an end to the process of apoptosis [120] and by binding of E7 to pBR, the expression of the necessary proteins for the replication of the DNA is stimulated [118]. Throughout the cancer progression, the integration of the host chromosome and viral genome causes a persistent level of E6/E7 [121] and the constant activity of E6 and E7 can augment the instability of the genome, create an aggregation of oncogene mutations, destroy more and more the control of cell growth, and sooner or later cause cancer (mostly cervical cancer) [122].

### Beta-glucan and cervical cancer

In our knowledge, beta-glucans have an anti-cancer property because of their immunomodulation role on T cells and antigen-presenting cells such as dendritic cells, macrophages, and B lymphocytes [87, 123, 124]. As a result, beta-glucans also might be effective in reducing cervical cancer progression through modulating both innate and adaptive immunity [123]. Several empirical studies have been conducted in order to investigate whether beta-glucans can be used as novel adjuvant treatment or prognosis approaches (Table 2).

**Table 2:** Functions of diverse beta-glucans and their application in cervical cancer

Beta-glucan	Model (In vitro, In vivo, Human)	Outcome (s)	Application in cervical cancer (Therapeutic / Prevention)	References
SPG	Human study	Promoting the recovery of T lymphocytes and natural killer cells	Treatment (adjuvant to radiotherapy)	[125]
SPG	Human study	Increasing the survival and recurrence time	Treatment (adjuvant to immunotherapy)	[126]
SPG	Human study	stimulating a rapid recovery of the immunologic parameters impaired by radiotherapy	Treatment (adjuvant to radiotherapy)	[127]
SPG	Human study	Increasing the survival and recurrence time	Treatment (in combination with irradiation therapy)	[128]
SPG	Human study	Increasing the number and secretion of TNF, IL-1 and IFN-gamma	Treatment	[129]
SPG	Human study	Augmentation helper T (Th) cell functions of pelvic lymph nodes	Treatment	[130, 131]
SPG	Human study	Increasing infiltration of Langerhans cells and improving local response to radiation treatment	Treatment	[132]
SPG	Human study	Augmentation of lymphocyte infiltration	Treatment	[133]
SPG	Human study	Augmentation of lymphocyte and Langerhans cell infiltration	Treatment	[134]
Curdlan	In vitro	More cytotoxicity and a broader distribution of loaded drug	Treatment (as drug carrier for epirubicin)	[135]
LBE	In vitro	Anti-cancer effects	Treatment	[136]
SCG	In vivo	Enhancing the hematopoietic response, recovering leukocyte population in peritoneal cavity	Treatment	[137]
PG101	In vivo	Induces the differentiation of progenitor cells to granulocytes and/or proliferation of the committed cells	Treatment	[138]
Zymosan	In vivo	Induction in the activity of peritoneal macrophages	Treatment	[139]
PBG	In vitro	Affects on both humoral and cellular immune response by increasing the cytotoxic and helper T cells and the release of IFN-γ	Treatment and prevention	[140]
Lentinan	*–*	Increasing drug efficacy	Treatment (combined with cisplatin and docetaxel)	[141]

Some efforts were made in the field of cervical cancer prevention and demonstrated that beta-glucans are also able to affect HPV infection. Stentella et al. [142] examined the carboxymethyl β-glucan (Colpofix®) gel on a group of people affected by CIN1. This case-control study demonstrated that Colpofix® has an anti-cervical cancer role in regression of CIN1. In 2010, two studies were conducted on the subject of “the beta-glucans’ influence on the HPV-correlated lesions in the genital area”. The first study found that beta-glucans are able to treat the lesions related to HPV infection [143] and the second study revealed the efficacy of beta-glucan treatment for HPV-CIN1 lesions [144]. These studies suggested that besides the anti-cancer effects of beta-glucans, they also have some effects on HPV infection, the main cause of cervical cancer.

Most of the recent researches on beta-glucans have been conducted on the sizofiran and its association with cervical cancer. Sizofiran or SPG is a beta-glucan that is derived from a mushroom called schizophyllun commune Fries [145]. The therapeutic anti-cancer impacts of sizofiran have been proven in murine and rats [146–148]. A study shows that SPG does its function though increasing the infiltration of T cells and Langerhans cells (which are one kind of the antigen-presenting cells for T cell responses) [134, 149] and so SPG can be used for the treatment of advanced cervical cancer to prolong the patient’s survival [133]. Nakano et al. [132] revealed that not only infiltration of Langerhans cells or ILC enhances T cell response also it can improve the local response to radiation treatment in cervical cancer. Shimizu et al. [130, 131] showed another function of SPG. They revealed that sizofiran is also able to enhance the function of helper T cells which are located in lymph nodes of pelvic in cervical cancer and additionally they observed an enhanced IL-2/IL-2R system. Chen et al. [129] discovered that SPG can elevate the secretion amounts of TNF, IFN-gamma, and IL-1 from peritoneal macrophages and thereby affect on the cancer progression. Furthermore, some papers are published about the usage of SPG as adjuvant therapy in the cervical cancer treatment. Hasegawa et al. [150] used a combination of radiotherapy and SPG as a treatment for cancer of the uterine cervix and observed that this adjuvant therapy leads to stimulating the cytotoxic function of macrophages and increasing the activity of natural killer cells. Okamura et al. [128] declared that using SPG can prolong the survival and recurrence time in these patients but its influence on different stages of the cancer is the same. Sizofiran is proven to cause a longer survival if used in combination with radiotherapy because it is able to develop a rapid recovery for immunologic parameters that radiotherapy destroys [125–127].

Curdlan is another polysaccharide in which some studies have been conducted on. It is a microbial beta-glucan that is derived from Agrobacterium spp. and is insoluble in water [151, 152]. Investigations showed that curdlan can play an inhibitory role against the infection of the AIDS virus and it also has some anti-coagulant functions in the blood. Besides all the roles discovered for curdlan, it appears to have low toxicity in vitro and in vivo [153, 154]. On top of that carboxymethylated curdlan or CMC is also known for its anti-tumor effects [155–157]. As well, Li et al. [135] synthesized cholesterol-conjugated carboxymethyl curdlan or CCMC which is a new kind of amphiphilic polymer and found that this polymer can be used in cancer treatment. Entrapping epirubicin or EPB which is a drug used in chemotherapy which in CCMC can dissolve it, lengthen its time of retention in the circulation of plasma, improve its therapeutic efficacy, and decrease its toxicity and so it has an importance in the cancer therapy as a novel carrier for epirubicin. Some other studies have demonstrated that also lentinan, a lentinula edodes-derived beta-glucan, can improve the efficacy of chemo and radiotherapy in many cancers including cervical cancer [158]. Ghosh et al. [136] also worked on effects of an ethanolic extract from Lenzities betulina named LBE on cervical cancer cell lines and observed anti-cancer effects against HeLa, CaSki, and SiHa.

Roopngam and collogues investigated the Pleurotus Sajor-caju-β-glucan polysaccharides or PBG and identified that PBG can affect on both humoral and cellular immune response by increasing the cytotoxic and helper T cells and the release of the IFN-γ. Therefore it can be used as a vaccine with HPV16E7 for the prevention and treatment of cervical cancer [140]. A trial examined a beta-glucan derived from edible mushroom Sparassis crispa named SCG and found that SCG can boost the hematopoietic responses and leukocyte population in peritoneal cavity so this beta-glucan can be a candidate for being used adjuvant to chemotherapy but further specific researches on cervical cancer are needed [137]. Jin et al. [138] explored the PG101, an extracted from Lentinus lepideus, and declared that this beta-glucan not only has anti-cancer effects (such as increasing proliferation of the committed cells) also can repair the damaged bone marrows, a result of chemo radiotherapy, and that is why it can be used as an adjuvant with chemo and radiotherapy but still. Nevertheless, further investigations are needed to show its effects on cervical cancer. In addition, beta-glucans extracted from yeast can have an enhancer role in the proliferation of the hematopoietic cells, and it can promote recovery of the leukocytes which are damaged because of the sub-lethal irradiation [159]. Nikulina et al. [139] used zymosan after treatment with actinomycin D, karminomycin and bleomycin in mice and observed that zymosan can cause an induction in the activity of peritoneal macrophages and thus, it might have therapeutic application in cervical cancer. Moreover, there are two alternative approaches for cancer treatment which previously have attracted a lot of attention including mAB-based therapy and using MDSC suppressors. The efficacy of beta-glucans has been significantly proven in both of these methods and hence, beta-glucans are counted as a potential therapy in many kinds of cancer. Considering the potential of both these two methods for being used generally in cancers (exclusive of the type of cancer), they are promising therapeutic approaches for cervical cancer as well. Nevertheless, further investigations are still needed to prove the efficacy of these methods in cervical cancer.

## Conclusions

Beta-glucans are known as immune system modulators which they can affect on a wide range cells including T cells, macrophages, monocytes, and antigen-presenting cells (APCs) like dendritic cells and thereby they can impact both innate and adaptive immunities. Recent studies with focusing on their influence on cytotoxic and helper T cells, APCs, inflammatory pathways, and oxidative burst (by using reactive oxygen species to destroy cells.) have revealed that they can also have some anti-cancer properties. Even so the main mechanisms in which these glucans function are inducing the APCs and T cells and thereby activating the anti-tumor immune system and subverting the suppressors of this immune system by stimulating the differentiation of MDSCs. It is also worth to mention that a study represented an antioxidant effect of oat beta-glucans [160] but further investigations are needed to prove their role in cancer prevention by scavenging free radicals. Likewise, a mAB-based therapy which is a medical progression into a new era of cancer treatment is only effective on tumor cells using adjuvant beta-glucans. The mentioned evidences have shown that sizofiran, zymosan, curdlan, PBG, and other beta-glucans can be employed in the prevention or treatment of cervical cancer. Generally, in cancer treatment, both chemo and radiotherapy have some side effects. One of the most important side effects of chemotherapy is neutropenia. Chemotherapeutic drugs disturb the process of the formation of the blood. Hence, chemotherapy has negative effects on the defense system of cancer patients and makes them susceptible to infections. Besides, radiotherapy has some side effects as well. It also leads to hematopoietic and immune damage. Thus, anemia, lymphocytopenia, thrombocytopenia, and granulocytopenia are the unpleasant consequences of radiotherapy [161]. A novel role of beta-glucans has been discovered in recovering hematopoiesis caused by injured bone marrow and so beta-glucans not only have anti-cancer impressions but also can be used adjuvant to common therapies for cancer to reduce their side effects in a cancer patient’s especially cervical cancer cases. Collectively, beta-glucans represent a novel promising therapeutic way for cervical cancer.

**Fig. 1:**
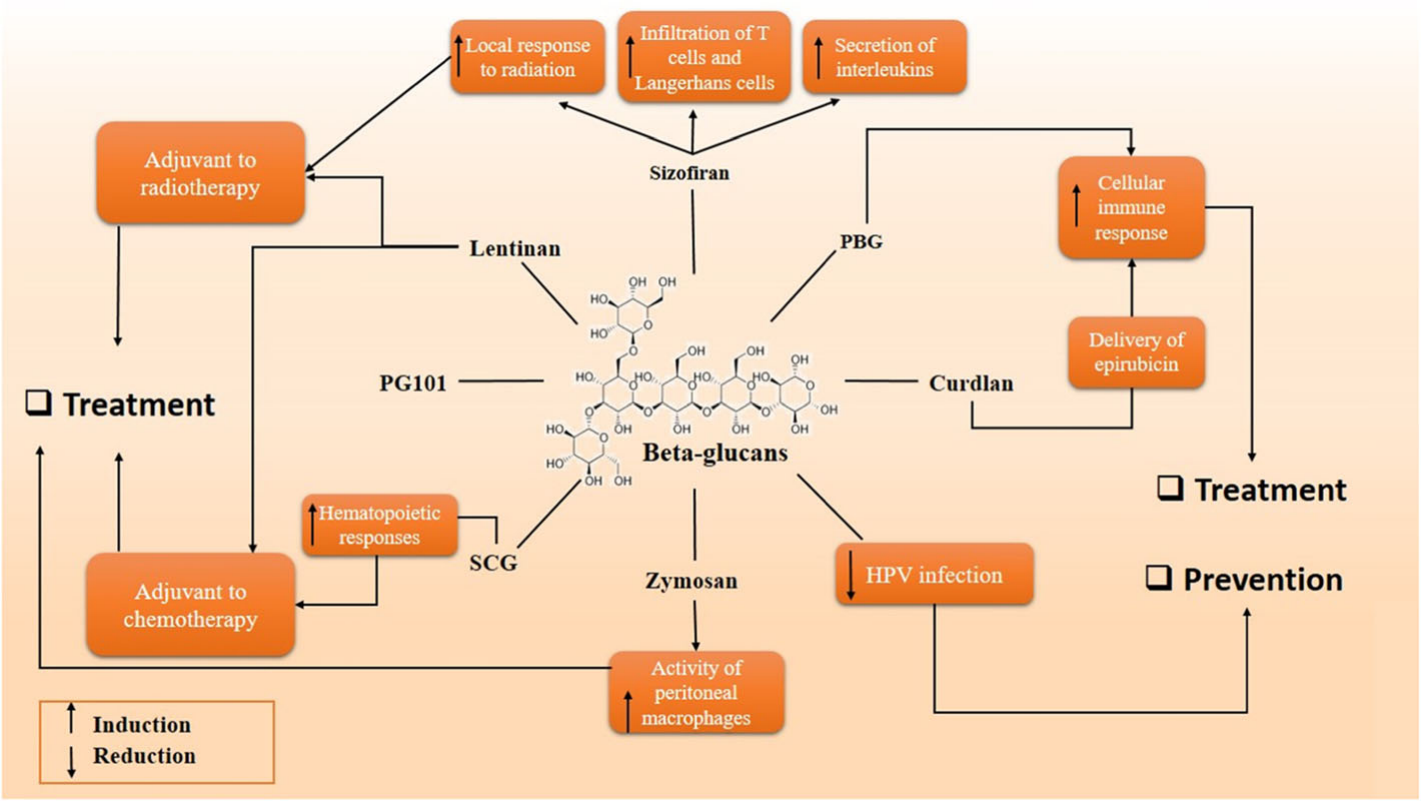
Schematic representation in targeting different pathways using beta-glucans as a novel therapeutic approach in the treatment of cervical cancer

Taken together, it seems that further investigations are needed on beta-glucans to prove their toxicity on cervical cancer cells, non-toxicity on normal cells, and their ability for carrying drugs to the cancer site. We suggest that some beta-glucans such as pleuran, chrysolaminarian, laminarin, and zymosan which are not properly taken into consideration in the cervical cancer therapy field, might be convenient candidates for their characteristics such as anti-inflammatory activities and anti-oxidant impacts (Fig. 1). Moreover, further studies on delivering chemotherapeutic drugs by schizophyllan and curdlan might give new insight into cervical cancer therapy. More explorations on the influence of beta-glucans on the CR3 receptor cervical cancer cells would open new horizons for replacing dangerous common therapies with non-toxic treatments. In the prevention point of view, there is a need for more researches on the mechanisms by which beta-glucans influence HPV infection and also a need for more researches on delivering HPV proteins by beta-glucans as a vaccine.

## Data Availability

Not applicable.

## Abbreviations

ADCC: Antibody-dependent cytotoxicity
BGR: Beta-glucan receptor
CCMC: Cholesterol-conjugated carboxymethyl curdlan
CDC: Complement dependent cytotoxicity
CMC: Carboxymethylated curdlan
CR3: Complement receptor3
CR3-DCC: CR3-dependent cytotoxicity
EPB: Entrapping epirubicin
HPV: Human papillomavirus
IL-1: Interleukin-1
IL-2: Interleukin-2
ILC: Infiltration of Langerhans cell
LPS: Lipopolysaccharide
mAB: Monoclonal antibody
MDSC: Myeloid-derived suppressor cells
PBG: Pleurotus Sajor-caju-β-glucan
PGG: Poly-[1, 6]-D-glucopyranosil-[1, 3]-D-glucoyranose
PMN-MDSC: Polymorphonuclear-myeloid derived suppressor cell;M-MDSC, monocytic-myeloid derived suppressor cell
pRB: Retinoblastoma protein
PRR: Pattern recognition receptor
TF: Tissue factor
Treg: Regulatory T cell
